# Editorial: The role of inflammation, stem cells and progenitor cells in cardiovascular repair

**DOI:** 10.3389/fcvm.2023.1195863

**Published:** 2023-04-18

**Authors:** Nanako Kawaguchi, Antonino Bruno, Prabhakara R. Nagareddy, Toshio Nakanishi, Murugesan V. S. Rajaram, Gaia Spinetti

**Affiliations:** ^1^ Department of Pediatric Cardiology and Adult Congenital Cardiology, Tokyo Women’s Medical University, Tokyo, Japan; ^2^ Immunology and General Pathology Laboratory, Department of Biotechnology and Life Sciences, University of Insubria, Varese, Italy; ^3^ Laboratory of Cardiovascular Research, IRCCS MultiMedica, Milan, Italy; ^4^ Department of Surgery, The Ohio State Unversity Wexner Medical Center, Columbus, OH, United States; ^5^ Department of Microbial Infection and Immunity, The Ohio State Unversity Wexner Medical Center, Columbus, OH, United States

**Keywords:** editorial, frontiers, cardiovascular medicine, inflammation, cardiovascular repair

Editorial on the Research Topic The role of inflammation, stem cells and progenitor cells in cardiovascular repair

This editorial features the collection of articles published in *Frontiers in Cardiovascular Medicine: The role of Inflammation, Stem Cells and Progenitor Cells in Cardiovascular Repair*. Myocardial infarction (MI) is the leading cause of death worldwide. After MI, immune cells, both tissue-resident and those recruited from the circulation, act to clear the dead cells and instruct the repairing process. However, the activated immune cells also cause chronic inflammation, which may aggravate the tissue damage, including aberrant healing finally resulting in fibrosis. Indeed, the role of inflammatory cells appears to be double edge: at first instance, inflammation acts as a host-dependent rescue/defense mechanism (in the acute phase); later on (in the chronic phase) inflammation turns into a damaging phenomenon for the host organism. These aspects are objects of the articles contributing to this collection that are briefly summarized in this editorial. Li et al. reviewed and discussed the two-sided roles of the cells involved in inflammation. Kumar et al. demonstrate biphasic kinetics of CD4^+^ T-lymphocytes after MI using animal models. Yang et al. identified *PFKFB2* as a key gene for the transition from acute to old MI in humans. The interaction between diabetes and MI is studied by Baumbach et al. who investigated CD34^+^/CXCR4^+^ cells in diabetic MI.

*Cardiac repair after myocardial infarction: A two-sided role of inflammation-mediated* by Li et al. Cardiac inflammation and the timely resolution of inflammatory processes are critical to prevent adverse cardiac remodeling and heart failure. After myocardial infarction, neutrophils first arrive at the damage site within 6–24 h followed by monocytes and macrophages, which contribute to myocardial cell death and injury. Then, monocyte, macrophages, and regulatory T cells are recruited and produce anti-inflammatory mediators and resolvins to promote cardiac repair. In this process, other cells such as fibroblasts can influence the immune cells by a paracrine effect of TGF-β. Neutrophils and macrophages have two-sided roles (pro-inflammatory and anti-inflammatory) depending on the cellular subtypes (N1 and N2 for neutrophils, M1 and M2 for macrophages). Interestingly, non-coding RNAs and epigenetic modifications regulate the fate of immune cells either pro-inflammatory or anti-inflammatory in the cardiac environment. They concluded that these anti-inflammatory drugs, non-coding RNAs, and epigenetic modulators could be the target for MI therapy.

*CD4^+^ T-lymphocytes exhibit biphasic kinetics post-myocardial infarction*. In this manuscript, Kumar et al. report preclinical data collected in mice that shed new light on the ongoing debate on the acute versus chronic effects of CD4^+^ T cells in post-MI physiopathological events. It is known that mice lacking CD4^+^ (global CD4^−/−^ animals) are defective in myocardial healing after acute myocardial infarction (MI) and show increased mortality (1). On the other hand, CD4^+^ T cells are important in cardiac remodeling during heart failure (HF) (2). Therefore, it is of high relevance to dissect the T-cell responses to wound healing (in MI) vs. tissue remodeling (in HF). The study presented in this article reveals that T-cell phenotype and activation state is different in MI vs. HF. In the HF model, the transmigration of all T-cell subsets (Th1, Th2, Th17, and Tregs) is bi-phasic during HF. Furthermore, authors introduced Rosa26-iDTR (inducible Diohteria Toxin Receptor) to CD4-cre mice and demonstrated that the depletion of CD4^+^ T cells at the second phase of transmigration significantly inhibited the LV remodeling and increase of end-systolic and end-diastolic volumes (EDV and ESV) during MI. Thus new studies dedicated to the associated molecular mechanisms are warranted.

*Identification of PFKFB2 as a key gene for the transition from acute to old myocardial infarction in peripheral blood*, Post-MI inflammatory and metabolic pathways play a significant role in deciding the fate of structural and functional properties of the injured heart to HF. However, it is unclear how the initial immuno-metabolic response during acute MI (AMI) evolves over a period of time (old MI; OMI) during the progression of HF. In this manuscript, Yang et al. provide valuable insights into the signaling pathways impacted by circulating immune cells during the transition from acute AMI to a more advanced OMI. Using Cibersoft, WGCNA, and Random Forest Model, they identified a key glyclosis gene, *PFKFB2* that seemed to associate with the differential distribution of immune cells. They found low expression levels of *PFKFB2* in peripheral blood cells from OMI samples compared to AMI samples. The authors suggest that PFKFB2 can help predict the distribution of immune cells during the transition.

The PFKFB2 gene encodes 6-phosphofructo-2-kinase/fructose-2,6-biphosphatase 2, an enzyme that is involved in both the synthesis and degradation of fructose 2,6 bisphosphate, a regulatory molecule that controls glyclolysis in humans. 6-phosphofructo-2-kinase/fructose-2,6-biphosphatase 2 has a 6-phosphofructo-2-kinase activity that catalyzes the synthesis of fructose-2,6-bisphosphate, and a fructose-2,6-biphosphatase activity that catalyzes the degradation of fructose-2,6-bisphosphate. This enzyme regulates fructose-2,6 bisphosphate levels mainly in the heart while a similar enzyme controlled by another gene regulates fructose-2,6 biphosphate levels in the liver and muscle. Thus, to identify this gene in leukocytes and associate its expression to the distribution of immune cells in OMI is interesting and novel. Previous studies have shown that AMI-induced hypoxia in cardiomyocytes activates HIF/Akt pathways and increases the expression of PFKFB2 which in turn regulates glycolysis and suppresses apoptosis (3). Besides, neutrophils and macrophages in the heart also show characteristics of enhanced glycolysis during AMI but the role of PFKFB2 in peripheral leukocytes during OMI stage is unclear (4). It is intriguing to note that PFKFB2 levels were also elevated in patients with no history of MI similar to AMI patients. These studies suggest that the PFKFB2 levels in leukocytes simply represent the “metabolic state” of the cell. Why PFKFB2 levels are decreased in immune cells during OMI is unclear. Thus, further studies are required to understand the specific role of this enzyme and study its implications on metabolic and inflammatory pathways during acute and choric stages of MI. Since the effect of various other risk factors such as smoking, age, gender, diabetes, etc. were not adjusted, it is premature to ascertain a functional significance to lower expression levels of PFKFB2 in leukocytes in chronic MI.

*A cohort study of circulating progenitor cells after ST-segment elevation and non-ST segment elevation myocardial infarction in non-diabetic and diabetic patients*.

The study by Baumbach et al. aimed at investigating whether MI severity and diabetes might interactively affect the migratory capabilities of CD34^+^/CXCR4^+^ progenitor cells, to determine if the migratory test can be effective in predicting cardiac outcomes. A longitudinal study was performed on patients with or without diabetes with a STEMI or NSTEMI. The endpoints were the measurement of circulating CD34^+^/CXCR4^+^ cells (by flow cytometry) and the determination of their migratory activities, *in vitro*, using samples collected at days 0 and 4 post days post-infarct. Cardiac function was monitored at 3 months using cardiac MRI. The population study included the following subjects: 50 patients with STEMI (16 with diabetes) and 21 NSTEMI (8 with diabetes). The authors observed that patients after STEMI had an increased frequency of circulating CD34^+^/CXCR4^+^ cells, compared with NSTEMI, with a further increased frequency of circulating CD34^+^/CXCR4^+^ in diabetes vs. non-diabetes subjects. In addition, the migratory capabilities of CD34^+^/CXCR4^+^ cells increased in STEMI vs. NSTEMI patients, suggesting migration *in vitro* is proportional to the extent of myocardial damage. Interestingly, the authors observed an opposite association between the CD34^+^/CXCR4^+^ cell migratory capabilities in patients with diabetes. Finally, the authors were not able to detect any statistically significant association between the *in vitro* CD34^+^/CXCR4^+^ cell migratory activities and MRI outcomes. The authors propose that monitoring CD34^+^/CXCR4^+^ may provide clinically useful information in ischemic cardiovascular disease.

*Bibliometric analysis of the inflammation in diabetic cardiomyopathy*, by Zhu et al. This study used bibliometric analysis, which searched the articles and reviews in the Web of Science (WOS) Core Collection using “inflammation” and “diabetic cardiomyopathy (DCM)” as search terms from 1 January 2001 to 28 February 2021, and visualization analysis and statistical analysis were conducted by Microsoft 365 Excel and VOSviewer 1.6.18. From that analysis, the publications of inflammation and DCM linearly increased in these two decades. They identified the leading countries, researchers, and journals. Inflammation in DCM was connected to the keywords such as inflammatory molecules (e.g., NF-κB), fibrosis molecules (e.g., TGF-β), and therapeutic drugs (e.g., empagliflozon). The data from these studies provide deeper insights into global trends in research related to the pathophysiology of DCM.
